# Reviving tradition-bound products: a case of value co-creation using rhetorical history

**DOI:** 10.1007/s11628-022-00504-w

**Published:** 2022-08-10

**Authors:** Chikako Ishizuka, Tseng Kuo-Che, Yasuyuki Kishi

**Affiliations:** Faculty of Economic Sciences, Niigata University, 2-8050 Ikarashi Nishi-ku, Niigata, 950-2181 Japan

**Keywords:** Service-dominant logic, Rhetorical history, Value co-creation, Tradition-bound products, Sake

## Abstract

This study explores the value co-creation framework to revive tradition-bound products using rhetorical history and service-dominant logic. This framework shows the effects of using historical significance to enable value co-creation in a new ecosystem by engaging consumers and local communities without eliminating their traditions. Existing studies merely discuss the methodology of a rhetorical emphasis on the authenticity of traditional industrial firms’ history to attract customers. This study explains the motivation to engage in value co-creation to transform tradition-bound businesses. Through these processes, businesses’ boundaries are thawed, and the customer becomes the advocate and thus, the driver of reviving tradition-bound products.

## Introduction

The threat of disappearing traditional, cultural, and property knowledge particularly in traditional handicraft industries is a growing problem (e.g., Shafi et al. 2021). In Japan, traditional industries that are closely related to life and culture have been declining since the end of WWII; however, the gross domestic product has been growing (Sasaki et al. 2021).

Firms have used rhetoric about their history to emphasize authenticity and attract customers to try and revive these industries but have not been very successful. In the study of rhetorical history (Suddaby et al. 2010), this method puts forth the differentiated competitive advantage of authenticity. For instance, firms strategically tell customers their story of history and culture to enhance their value (e.g., Mora and Moscarola 2010; Mora and Livat 2013; Lee and Shin 2015). However, this type of rhetorical history comes within the ambit of value provision, and there has been insufficient discussion from the viewpoint of value co-creation.

It has long been noted that consumers value the consumption process and the consumption experience itself (e.g., Holbrook and Hirschman 1982; Pine and Gilmore 1999). In recent years, technological innovation has changed the focus of consumption from material to experience (Morewedge et al. 2021). This transformation indicates the importance of a shift from a goods-dominant (G-D) logic, which is based on exchange value and places products and firms at the center, to a service-dominant (S-D) logic, which places services at the basis of exchange and value being co-created (Vargo and Lusch 2004, 2008b). The importance of this paradigm shift from G-D logic to S-D logic and the role of value co-creation in the market are well discussed in the literature (e.g., Prahalad and Ramaswamy 2004; Vargo and Lusch 2004, 2008a, b; Vargo et al. 2017). However, what motivates consumers to engage in value co-creation or how a company’s value is co-created under constraining conditions, such as tradition-bound products, is not sufficiently clear.

To fill the research gap, this study explores the theoretical framework of value co-creation that can revive tradition-bound products using rhetorical history (Suddaby et al. 2010) and S-D logic (Vargo and Lusch 2004, 2008a, b), integrating two academic concepts used in organizational and marketing research. To this end, we consider a case study of a brewery in Japan that has been operating for more than 470 years and has launched a new sake project, which has benefited from the masterful use of rhetorical history. Furthermore, it has also built a new ecosystem that combines factors, such as the tradition of sake culture, funding platforms, local craftsmanship, and new customers outside of local areas. Consequently, this project has succeeded in involving customers, which has helped develop a specific fan community of sake.

Furthermore, this theoretical framework helps us understand how to overcome the problems that firms face in effectively using rhetorical history and avoiding its negative use through the euphemistic concealment of inconvenient facts, as pointed out in previous studies (e.g., Aeon and Lamertz 2021). It also provides useful insights for storytelling as well as retro-marketing that seeks to connect brands and consumers more deeply and emotionally (e.g., Pİr 2019; Gajanova and Zdenka 2020).

The rest of the paper is organized as follows. The next section provides a brief overview of the consumption of a tradition-bound product, like sake. The third section provides the theoretical background and research question. In the fourth section, the research method is described. The fifth section describes the details of the case study. The sixth section provides a discussion of key implications and conclusions. The last section describes the limitations of the study and future research directions.

## Consumption of tradition-bound products and the sake business

In Japan, the number of people engaged in traditional industries decreased from 288,000 in 1979 to approximately 62,000 in 2017, and the production value declined from 540 billion yen in 1983 to 96 billion yen in 2017 (Association for the Promotion of Traditional Craft Industries 2021).

The growth and change in the consumption of basic consumer goods, such as alcoholic beverages, are closely linked to the process of change in daily life and income associated with economic development (Francks 2009). Similar to other traditional products, such as Japanese sweets and green tea, sake consumption has been declining since the period of rapid economic growth (1950s–1970s), as dietary habits and living standards have changed (Matsuda 1981). Due to the increasing consumption of new imported alcoholic beverages since the first Tokyo Olympics in 1964, sake consumption is now at a third of its peak in 1973 (Shima 2007; JNTA 2019).

Facing continuously declining sales, many breweries are struggling to revive their business. For instance, some breweries are taking on the challenge of producing local but authentic sake to preserve the culture and belief of “brewing for the local community” (e.g., Sumihara 2004). Some breweries are continuously striving to achieve a balance between tradition and new technology (e.g., Bouzdine-Chameeva et al. 2009). Furthermore, many breweries are expanding beyond their existing sake business (e.g., Xia and Donzé 2021).

Many alcoholic beverages, such as wine and beer, have greatly influenced the economy and civilization of human society for a long time (Cabras and Higgins 2016). Similarly, sake is not only rooted in Japanese culture and history (e.g., Sakaguchi 2007; Sasaki and Sone 2015) but has also played a role in helping people socialize beyond just getting drunk (Francks 2009). For instance, drinking sake with *ochoko*, a small ceramic cup, has a cultural element that cements social connections. There is also an expression called “tasting sake,” which indicates a “coolness” distinct from the consumption of the drink itself (Francks 2009). In 1980, Masayoshi Ohira, the then Prime Minister, said, “Sake is the national drink, especially when entertaining foreign guests” (Sato 2013). Therefore, the sake industry’s continuous decline is a serious concern from the economic, cultural, and social perspectives.

## Theoretical background and research question

### Value co-creation based on S-D Logic

Several streams of value-related research have focused on the contextual and experiential nature of value creation and determination and have shifted their primary interest to the importance of value-in-use rather than exchange value (Vargo et al. 2017). The hedonic nature of consumption, with its emphasis on value-in-use, has long been noted (e.g., Holbrook and Hirschman 1982), along with how consumers value the experience of consumption itself (Pine and Gilmore 1999). In recent years, owing to technological innovation, experiences are replacing material goods as the focus of consumption (Morewedge et al. 2021).

Value and value creation processes are seen from the perspective of consumer experience and not from product- or firm-centric views; the interaction between firms and consumers has shifted toward value creation and value extraction (Prahalad and Ramaswamy 2004). This shift represents the transition from G-D logic to S-D logic (Vargo and Lusch 2004, 2008b), in which services are the basis of exchange and value is co-created. The application of such a conceptual framework has changed the conceptualization of innovation from an output exchanged within a dyad to a new process of value co-creation (Vargo et al. 2020).

S-D logic is based on the fundamental proposition that all activities are considered as services, that both the company and customer create value, and that value is co-created interactively (Vargo and Lusch 2004, 2008b). Thus, it stands in contrast to the conventional product-centered logic that assumes that a company creates value, which is embedded in products provided unilaterally by the company to customers. Service is defined as “the application of professional competence (i.e., operant resources: knowledge and skills) through action, process, or performance for the benefit of another entity or the entity itself” (Vargo and Lusch 2008b). In contrast to these macro concepts, from a micro perspective, customer engagement occurs through an iterative process of value co-creation and contributes to the development and co-creation of customers’ personal and interpersonal operant resources (Brodie et al., 2011; Hollebeek et al., 2019).

In the S-D logic paradigm (Vargo and Lusch 2004, 2008b), experiences are created with other people in a social context, leading to the concept of “service ecosystems” to identify the flow of mutual service provision (Vargo and Lusch 2016). Such service ecosystems are “systems of resource-integrating actors connected by shared institutional logic and mutual value creation through service exchange” (Vargo and Akaka 2012, p. 207). They are further defined as “a relatively self-contained, self-regulating system of resource-integrating actors” (Vargo and Lusch 2014).

In contrast to the traditional view of the customer as a passive recipient of brand-related information, S-D logic emphasizes that value co-creation must always include the beneficiary, that value is co-creative, and that the decision is always independently and phenomenologically determined by the beneficiary.

Meanwhile, S-D logic is evolving toward a general theory of value co-creation as a social objective, not just for a particular subset of social activities (Vargo and Lusch 2016). The application of S-D logic has transcended to reconcile the tensions and paradoxes within existing ideas of a new definition of services and value co-creation (Vargo et al. 2020). In addition, customers voluntarily invest their cognitive, emotional, behavioral, and social knowledge and skills to interact with a brand in the service system (Hollebeek et al. 2019), and participation in value co-creation increases participants’ subjective well-being (Hughes and Vafeas 2021).

Thus, knowledge about the relationship between companies and customers, the benefits and perceptions of customer participation, and value co-creation have been created. However, it is not sufficiently clear how companies can shift from G-D logic to S-D logic for value co-creation with consumers; what motivates consumers to engage in value co-creation; and how value is co-created under constraints, such as tradition-bound products. In light of the above discussion, this study focuses on the concept of rhetorical history as an explanation for the formation of motivation for actors to participate in value co-creation.

### Rhetorical and strategic use of history

From an orthodox and objective perspective, history considers events and facts as things that have occurred and are difficult to change (Yin 2003; Suddaby and Foster 2017). In contrast, the social constructionist perspective sees histories not merely as comprising dead things in the past, but as sources that can be used rhetorically and strategically in management to lead the present to the future in the desired direction (Garud et al. 2010; Foster et al. 2017; Suddaby and Foster 2017). Regarding the concept of rhetorical history, Lamertz et al. (2016, p. 819) asked the profound question, “if the past can be a source of competitive advantage and past heritage can be successfully used to create new ventures and collective identities, what makes some organizations better than others in recycling the past for present usage?” Furthermore, Foster et al. (2017) systematically classified types of identification and audience, enabling scholars to examine the rhetorical and strategic use of history (Table 1).

**Table 1:** Effects of rhetorical history

	Audience		
Identification		Internal	External
	Uniqueness	Identity	Authenticity
	Similarity	Culture	Legitimacy

Source: Foster et al. (2017)

In the instance of sake brewery storytelling, *authenticity* can be developed when breweries impress their unique image upon external audiences, such as by emphasizing their traditional customs or culture rooted in industrial history to attract domestic and inbound foreign tourists (e.g., Mora and Moscarola 2010; Mora and Livat 2013; Lee and Shin 2015) and retro-marketing (e.g., Pİr 2019; Gajanova and Zdenka 2020). In the case of fine wine brands, *authenticity* can also be built by holding ceremonies associated with regional histories, traditional cultures, or myths to enhance their sincerity (Beverland 2005). In contrast, the Ontario fine wine industry sought *legitimacy* to enter a global institutional framework by repudiating its own previous illegitimate local history and appealing to the similarities in the global wine market (Voronov et al. 2013). When an organization shares its uniqueness with internal audiences, it can emphasize its centrality and distinctiveness and build an *identity* (Albert and Whetten 1985). However, when the similarity of the connection between social, religious, or organizational values is shared with internal audiences as historical narratives, a brand can build an organizational *culture* by appropriately selecting employees and adopting strategies suited to that organizational culture (Foster et al. 2017).

Thus, existing discussions of rhetorical history have primarily been concerned with one specific effect achieved by its use in one case or one pattern. For instance, authenticity as a specific effect of the strategic use of history on external audiences was discussed in detail in the case of Lee and Shin (2015). However, the strategic effects resulting from the rhetorical use of history in traditional industries with long histories may simultaneously affect external and internal audiences and vice versa.

Prior rhetorical history research has not discussed the changes in organizational boundaries resulting from the strategic use of history in detail, overlooking the possibility of external audiences becoming internal and vice versa due to changes in perceptions and empathy for specific historical meanings. From this perspective, it is necessary to re-think the existing concepts of external and internal audiences that may be “relative external audiences” and “relative internal audiences,” rather than to fix them completely. In other words, strategic use of history can potentially blur the line between an organization's external (i.e., customers in this study) and internal position.

### Positioning of this study

The directions of development of the social status of traditional products are identified as horizontal and vertical development (Delmestri and Greenwood 2016). A horizontal categorical dimension means the product category that allows us to know the order of its social status in comparison with other products, and a vertical categorical dimension means that social status is recategorized by endogenous forces within existing mature categories.

The effect of vertically developed status through a shift in social image can be confirmed in some specific cases of Champagne by respecting traditional methods (Guy 2003) and by educating consumers about tradition and manners (Dion and Borraz 2017); these vertical developments are based on a premise that companies provide value to consumers. These arguments are based on the traditional G-D logic, which assumes that companies with resources and narratives create value that improves products’ status and that the value embedded in products as objects is provided one-way by companies to customers (Vargo and Lusch 2004).

Meanwhile, Lee and Shin (2015) argued that the sake industry can be revitalized by drawing on the industrial history and brewing culture of sake breweries as resources and appealing to consumers and tourists through storytelling. As traditional products are embedded in history and culture, which can be resources for gaining competitive advantage, it is strategically important to improve their status in vertical development.

By contrast, typical horizontal development in sake, through proximity in mannerisms related to its consumption to those of wine, which dominates the global market, was specifically described by Wang (2019). While sake consumption declined by a third by 2017 compared to its peak in 1973, premium sake’s share in total sake sales increased from 15 to 46%, showing a shift toward higher-end sake consumption (JNTA 2019). Wang (2019) attributed this trend in sake consumption to the shift from functional consumption to hedonistic consumption, meaning that the consumption of sake is transforming from “getting drunk” to “enjoying sake.” Wang (2019) further suggested that sake reformation involves breaking away from traditional sake culture to market it to young urban consumers. For example, she presented the challenge of gaining legitimacy in the global market through sake nouveau and sake terroir, mimicking the context of wine (Wang 2019). Although there is an existing term in traditional sake culture called “Shinshu” (i.e., new sake), meaning the first brewed from rice harvested that year, the concept of sake nouveau incorporates a term borrowed from wine culture. It tries to refine sake culture using the wine context. Sato and Kohsaka (2017) argued for the need to brand sake as a terroir, also taking inspiration from the wine industry. In the concept of sake terroir, some sake brewers, especially those of premium sake, are also rice farmers, changing sake culture from relatively placeless to place associated (Wang 2019: 84–86). It can be said that the Japanese traditional sake industry should take inspiration from the wine industry to enhance its social status in the global market.

The suggestion to capture knowledge from outside of existing cultural meanings can be considered a horizontal development in traditional industry, similar to the implication of Wang (2019). Sato and Kohsaka (2017) advocate sharing the local history of production and reconstructing the emotional relationship between producers and consumers, pointing to the perspective of value co-creation between them.

This study examines value co-creation cases that vertically promote the market for tradition-bound products, which has been overlooked in existing studies that focus on the vertical development of G-D logic or on the horizontal development of S-D logic. Further, this study develops and proposes a new framework based on the integration of S-D logic and rhetorical history (Table 2).

**Table 2:** Positioning of this study

		Status development of sake	
		Vertical	Horizontal
Basic premise of value creation	Service-dominant logic	This study	e.g., Sato and Kohsaka (2017)
	Goods-dominant logic	e.g., Lee and Shin (2015)	e.g., Wang (2019)

### Research objective

Rhetorical history can be described as the rhetorical and strategic use of facts, regardless of how they occurred in the past, and is an important managerial resource to achieve specific objectives; inform certain, but relative, audiences; and share certain meanings. While previous studies have implicitly demonstrated that the rhetorical use of history helps in developing products’ status, they have focused only on G-D logic. It is not adequately clear how companies can shift from G-D to S-D logic and shift to value co-creation with consumers within the constraints of traditional products and other factors. Moreover, changes in organizational boundaries due to the strategic use of history have not been discussed in detail in past studies. Existing rhetorical history studies have considered organizational interiors and exteriors fixedly and have primarily focused on one specific effect achieved by the rhetorical use of history in one case or one pattern, with insufficient discussion of changes in organizational boundaries.

In other words, rhetorical history has shown its potential to promote traditional industries; however, the motivation to overcome the constraints of traditional industries and engage consumers in value co-creation is not well understood. Clarifying this research gap will lead to a better understanding of historical value co-creation in products bound by tradition. In light of the above, our research objective is integrating the two conceptual frameworks into a complementary conceptual framework that makes rhetorical history a result of co-creation of value rather than an exchange of value.

Our research question is as follows. How can value co-creation be achieved among entities by the rhetorical and strategic use of historical meaning in a traditional industry?

## Method

### Case study

We used the case study approach proposed by Yin (2003) to investigate Kayoi. According to Yin (2003), when researchers ask “why” and “how” questions about the research subject that is out of their control, case study and history become two appropriate research designs. The case study research design includes specific practical research tools to approach the current and accessible research subject, such as interviews or participants’ observation. However, history is an appropriate research design when researchers intend to approach subjects that occurred in the “dead” past and can only be investigated through secondary data.

To clarify the research question mentioned at the end of the theoretical background, we used the case study research design to examine the project of Yoshinogawa Kayoi, which is freshly invented by Yoshinogawa and bound in the specific history and culture of the Japanese sake brewing industry to co-create value with stakeholders. Although the design of multi-case studies can increase the validity of theory building (Eisenhardt 1989) and has a high degree of both external and internal validity (Bhattacherjee 2012), we considered Yoshinogawa Kayoi as a specific case that can deeply examine and discuss the connection between experiential economy, value co-creation, and rhetorical history. We conducted multiple face-to-face interviews and several email exchanges with the chief executive officer (CEO) of Yoshinogawa Corporation, the manager of the strategy department, and the brewer of Yoshinogawa. We also obtained a significant volume of data from consumer questionnaires on the 2020 Kayoi project from Yoshinogawa Corporation. Using KH Coder (Higuchi 2016), an open-source linguistic software to eliminate arbitrariness, co-occurrence network analysis demonstrated the impact of Yoshinogawa Kayoi through text mining analysis of customer attributes and comments.

### Research subject: Yoshinogawa Corporation

In this study, we focus on a new business project conducted by a sake brewery, Yoshinogawa Corporation, which has been brewing sake for over 470 years. This project has succeeded in the value co-creation process by bringing a new ecosystem into the sake market.

Yoshinogawa Corporation was founded in 1548 and is in Miyauchi. It is famous for its history of brewing sake, soy sauce, and miso and currently has about 80 employees. “Yoshi,” refers to the first name of the owner’s mother; “Gawa,” the other part of the firm’s name, means river in Japanese and particularly refers to the Shinano River, the longest river in Japan—running through the Niigata Prefecture. Further, water is the lifeblood of sake. In the sake brewing process, the brewery uses local rice and water that melts from the snow that piles up in the chain of mountains in this region and is collected from the Shinano River.

Although Yoshinogawa introduced and started the large-scale brewing production system in the mid-1950s, they have been working continually with the belief that “mechanization but not automation” is crucial for their sake production process. Daiginjo, the highest class of sake, is still brewed only by hand. Yoshinogawa is the fifth largest sake producer in Niigata Prefecture and the 30th largest in the overall domestic market. Yoshinogawa won the first prize in the Kanto-Shinetsu Regional Taxation Bureau five times and won the Kura Master in France in 2018, 2019, and 2021.

## Case of Yoshinogawa Kayoi

### Background of Yoshinogawa Kayoi project

**Fig. 1:**
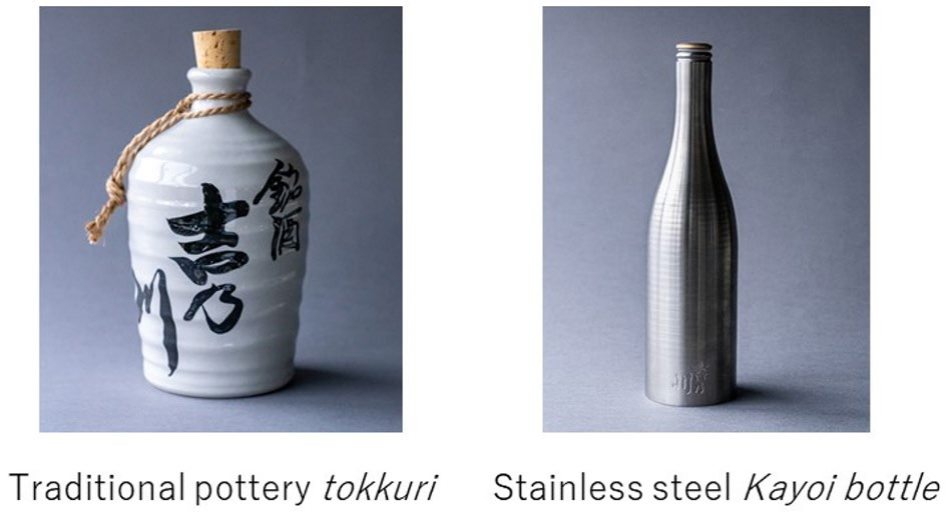
Evolution of the bottle through the fusion. Source: Ishizuka et al. (2021); approved by Yoshinogawa Corporation

The idea of Yoshinogawa Kayoi originated in the Edo period (1603–1868) with the concept of “*Kayoi-tokuri*.” During that period, there was a consumption system of selling sake, wherein it was poured into a *tokuri* with the store name or trademark written on it (e.g., left of Fig. 1) and given to customers. Ordinary people used this system called Kayoi, which means “going and coming back,” wherein they would go buy sake and bring it back in their own *tokuri* or cedar barrels (Imayasu 1980). When the *tokuri* was empty, customers would return it and pay to have it refilled. At that time, pottery bottles were the most common type of bottles for *tokuri*; however, Yoshinogawa Kayoi has transformed them into stylish stainless-steel bottles with excellent durability (Fig. 1). This is to ensure that the bottles are strong enough to be sent from the brewery to the customer’s home using the modern delivery system.

Prior to this project, the company had remodeled the brewery’s facilities to offer consumers a variety of brewery experiences. However, the CEO was still wondering whether consumers would consume sake despite having an impressive experience at the brewery. Through his Twitter interactions with customers, he had learned that they were drinking Yoshinogawa on their special days. This led him to think that “no snowflakes fall in the same spot, each of them has their own special day” (an “extraordinary day”). Nevertheless, he experienced a barrier: “Selling special sake through a sales channel where anyone can buy it at any time makes it less special.”

Therefore, he was searching for a way to make Yoshinogawa available to his customers on their special occasions so that they continue buying it. At that time, the CEO happened to see a product called “Glass to last you a lifetime at a department store.” It is usually challenging to transform glasses and ceramic mugs, which break easily, into something that will last a lifetime. However, the CEO was inspired by the concept of “connecting artisans and customers” embedded in the product. Yoshinogawa Kayoi was created with this belief. Eventually, the brewery rhetorically used the traditional custom of this concept to offer a new product and service to impress costumers. The brewery revived this traditional custom in the modern era using stylish and durable stainless-steel bottles, involving local metal manufacturing companies and using crowdfunding as a platform for securing funds.

### Fusion with local craftsmanship: building a new ecosystem

The CEO asked the President of Sasage Industry Co., Ltd., who is well known for his technical skills and craftsmanship, to make the Kayoi bottle. The President of Sasage sympathized with Kayoi’s concept and readily agreed to manufacture stainless-steel sake bottles, which is not technically easy (right panel of Fig. 1).

Yoshinogawa explained the concept of Kayoi: “Kayoi is a new relationship between a sake brewery and its customers, born from the combination of community’s proudest artisanal skills: sake brewing and metal crafting.” This stainless-steel bottle also won the Grand Prize from the Minister of Economy, Trade, and Industry at the Japan Tsubame Industrial Design Competition 2020.

### Taking advantage of the new platform

After creating the product, Yoshinogawa launched the Yoshinogawa Kayoi project using the Makuake crowdfunding platform in June 2020. The company set a low goal of 500,000 yen because many other breweries were running campaigns and they were concerned that their company would be left out. However, the campaign met its goal on the second day; within a week, applications from approximately 300 people were received and finally, the target of 5 million yen in funding was achieved.

The idea behind the Yoshinogawa Kayoi service is that if you buy one stainless-steel “my bottle” with a lot number, you can taste the premium sake brewed by Yoshinogawa at least once in the traditional but renewed system of “kayoi.” The number of Kayoi increased with the price of an application. For example, a plan with an application price of 35,000 yen allowed consumers to taste four special types of sake in four Kayoi, plus an additional set of three premium sake bottles.

In this way, the project created a unique “emotional experience” for customers. By delivering special sake directly to customers on their special day, Yoshinogawa would be with them on their special occasion and have them remember the product and experience. In addition, it enhanced the direct communication between the brewery and consumer through several visits and owning an exclusive bottle of Yoshinogawa. These factors developed a fan base, a shared brand, and a sense of values. In this way, Yoshinogawa co-created value with customers and increased its mindshare through direct connections. Furthermore, using this platform, the brewery was able to gain support from consumers who had no contact with Yoshinogawa.

### What motivated consumers to participate in the Kayoi project?

The Yoshinogawa Kayoi initiative gained 320 customers after using Makuake as its crowdfunding platform. According to a survey conducted by the brewery, about 50% of these 320 customers learned about the project from the Makuake website and supported it.

As per the data, 8% of customers were in their 20 s, 24% in their 30 s, 33% in their 40 s, 24% in their 50 s, 9% in their 60 s, and 2% in their 70 s or older group. Yoshinogawa and the wider industry succeeded in attracting the younger generation in their 20 s and 30 s, which was perceived as an industry challenge. In addition, participants came from 38 prefectures, showing the geographical spread of the event: 27% were from Tokyo and 17% were from Niigata Prefecture, where the brewery is located. The Yoshinogawa Kayoi initiative encouraged geographic and demographic expansion of the customer base, which would have been difficult for Yoshinogawa to acquire on its own.

Yoshinogawa Corporation sent a questionnaire to Kayoi project participants when they returned the bottles during the second round (in October 2020). The brewery received responses from 169 participants. Customers were asked about their experiences of drinking the brewery’s sake and their reasons for participating in the Kayoi project. Table 3 shows the questionnaire sent by Yoshinogawa Corporation to the participants and their answers.

**Table 3:** Questionnaire and answers for Yoshinogawa Kayoi project (*n* = 169)

Questionnaire	Answer choices	Results
1. What did you find attractive about the new Kayoi?	a. Historical content of Kayoi b. Bottle design c. Taste of sake d. Organized by Yoshinogawa	45% 29% 10% 16%
2. What do you think about the price of Kayoi?	a. High b. Fair c. Cheap	16% 82% 2%
3. Do you usually drink Yoshinogawa?	a. Drink b. Sometimes c. Do not drink	19% 50% 31%
4. What type of sake do you like best?	a. Junmai-shu b. Ginjo-shu c. Honjozo d. Ordinary sake	51% 37% 6% 6%
5. What kind of services or options would you like to see at Kayoi in future?	This is not a multiple-choice question. Please write your answer in words	Figure 2
6. Please feel free to write anything else	This is not a multiple-choice question. Please write your answer in words	

Source: Compiled by the authors; permitted by Yoshinogawa Corporation

**Fig. 2:**
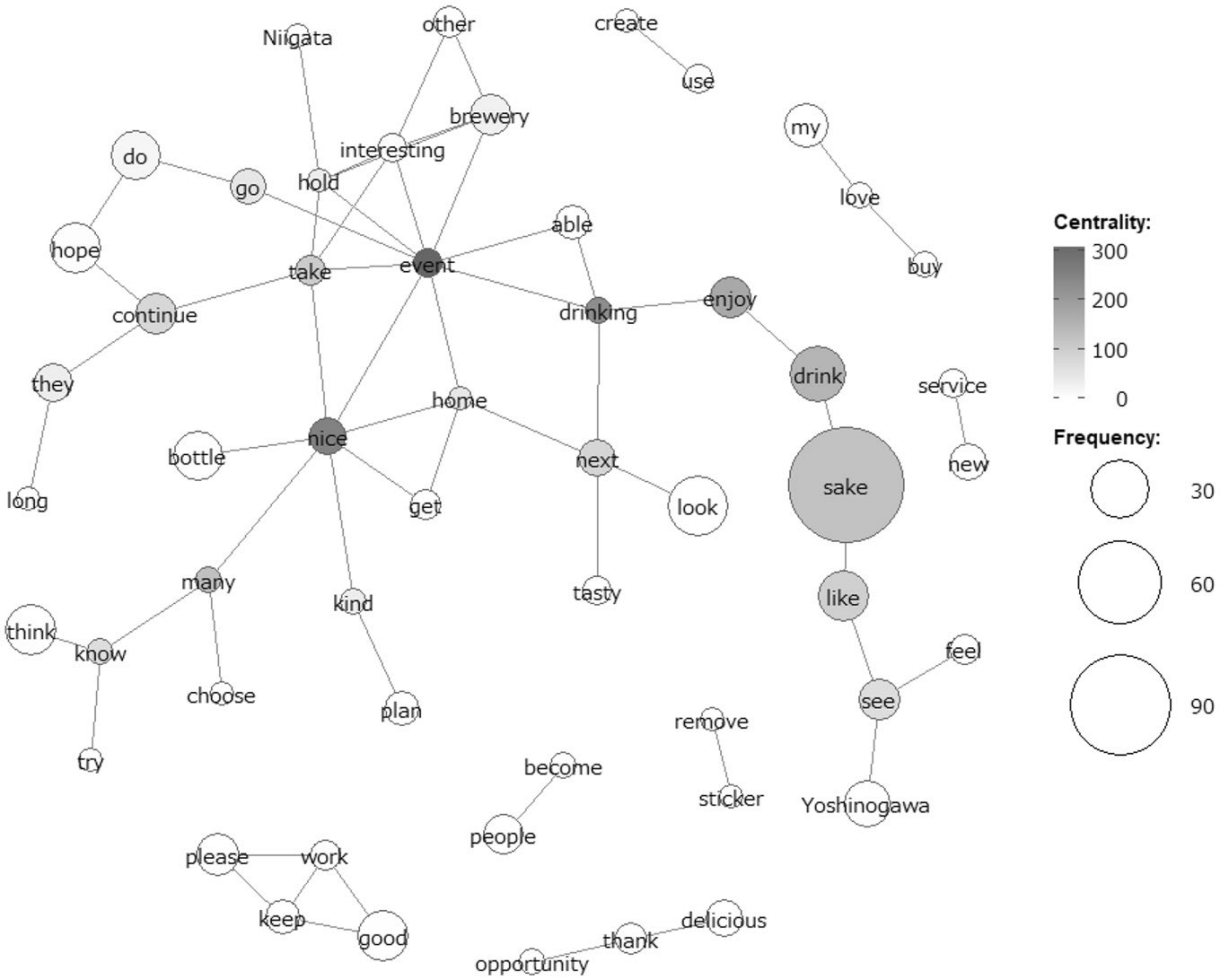
Results of co-occurrence network analysis of Kayoi customers’ comments

The most notable result of Yoshinogawa Kayoi was the development of fan consciousness among customers through their participation in this project. The most common reason for supporting this project was “historical content of Kayoi” (45%). The next most common reason was “bottle design” (29%). Only 10% respondents mentioned the taste of sake itself, clearly indicating that they valued the experience over the product’s taste (Table 3). Regarding the customers’ experience of drinking the sake from the brewery, 19% said they drank it all the time, 50% said they drank it occasionally, and 31% said they never drank it (Table 3). In other words, there is no underlying G-D logic that says they participated because they like sake. These results indicate that customers did not participate in this project just to buy sake; rather, they placed more emphasis on the historical custom of Kayoi, which is the project’s key success factor. Later, a community called “Kayoi-ally” was established by the project’s participants.

After responding to the multiple-choice questions, the participants were asked what they expected from Yoshinogawa Kayoi in the future and were given a free-text format field for their open-ended answers. All free-text descriptions obtained from the 169 participants were subjected to text mining to search for common preferences and orientations based on frequently occurring words and their connections. Co-occurrence network analysis using KH Coder (Higuchi 2016) confirmed the frequent words and their connections in written text. Co-occurrence is a network diagram in which extracted words that appear in the same paragraph are nodes and lines (links) connect them. The extracted words connected by the lines are co-occurrence relationships (used in the same paragraph). The diagram is most meaningful when considering the lines connecting the extracted words, rather than considering their placement. Color coding is based on mediation centrality in network analysis; darker colors correspond to higher mediation centrality. Between centrality is an indicator of the degree to which extracted words connect with other extracted words and shows the degree to which each node (frequent words in this study) is central to the network.

The analysis result was a co-occurrence network of the top 60 words extracted from 252 sentences, with a minimum frequency of five or more occurrences (Fig. 2). The analysis excluded interrogatives, conjunctions, and articles. The size of the circle represents the frequency of occurrence, and words that co-occur in the same context are depicted in the same subgraph.

The analysis shows that the words with the highest betweenness centrality are event, nice, drink/drinking, enjoy, sake, continue, next, and like. These have higher betweenness centrality than “brewery” and “Yoshinogawa.” As shown in Fig. 2, the context, understood from word connections, indicates that Kayoi’s style is empathetic. The style was created using a rhetorical history that relies on the national DNA and is, therefore, empathetic. In addition, words like “event” and “home,” which are usually separated, co-occurred as words with the highest intervals of centrality. This shows that the CEO succeeded in developing links between the extraordinary (special events) and the ordinary (home) that he was aiming for.

## Discussion and conclusions

### Theoretical implications

**Fig. 3:**
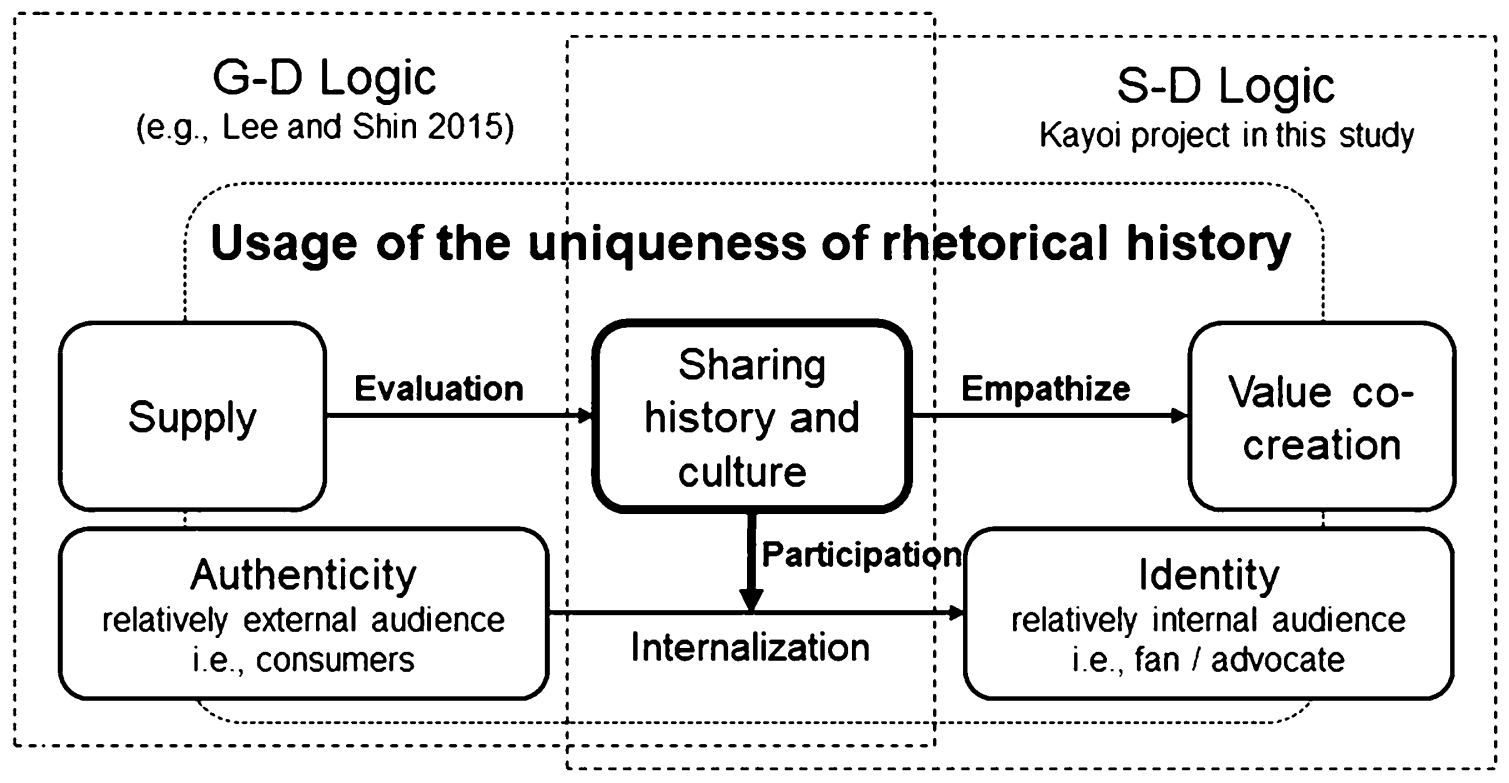
Value co-creation framework using rhetorical history

This study showed how companies and customers transition to value co-creation under constraints, such as tradition-bound products, and how the motivation to participate in value co-creation are formed. We also focused on—and explained changes in—organizational boundaries, which have not been adequately discussed in existing rhetorical history studies. The study conceptually explained how tradition-bound products and their services are transformed from G-D to S-D logic by internalizing value and sharing experiences and identities (Fig. 3).

The use of rhetorical history can motivate value co-creation (Vargo and Lusch 2004, 2008b) by gaining empathy from each actor linking the service ecosystem (Vargo and Lusch 2016). This suggests motivation to promote consumer participation, which has not been adequately discussed in existing research. In the case of Yoshinogawa Kayoi, the consumers themselves participated in the new ecosystem, deepening their connection and co-creating value through empathy for the history and culture of sake. The strategic use of shared histories resulted in dyadic interactions among actors who built authenticity, formed service ecosystems and communities that created identities, and became a driving force to develop the social status of traditional products and services further vertically.

This indicates that assets accumulated in the past (Kayoi culture) can become a source of significant competitive advantage. However, it is more important to create a new relationship with customers by giving them experiential value (Pine and Gilmore 1999). This requires using assets accumulated in the past in a new and innovative manner and not merely as they are. Customers internalize *authenticity* through their participation in value co-creation. The internalization of *authenticity* transforms them from merely being a passive consumer to a fan or advocate, and they finally achieve a co-building of identity (Fig. 3).

We explained the theoretical framework of motivating co-creation using a case of reviving tradition-bound products that have faced declining consumption due to changing lifestyles.

### Managerial implications

**Fig. 4:**
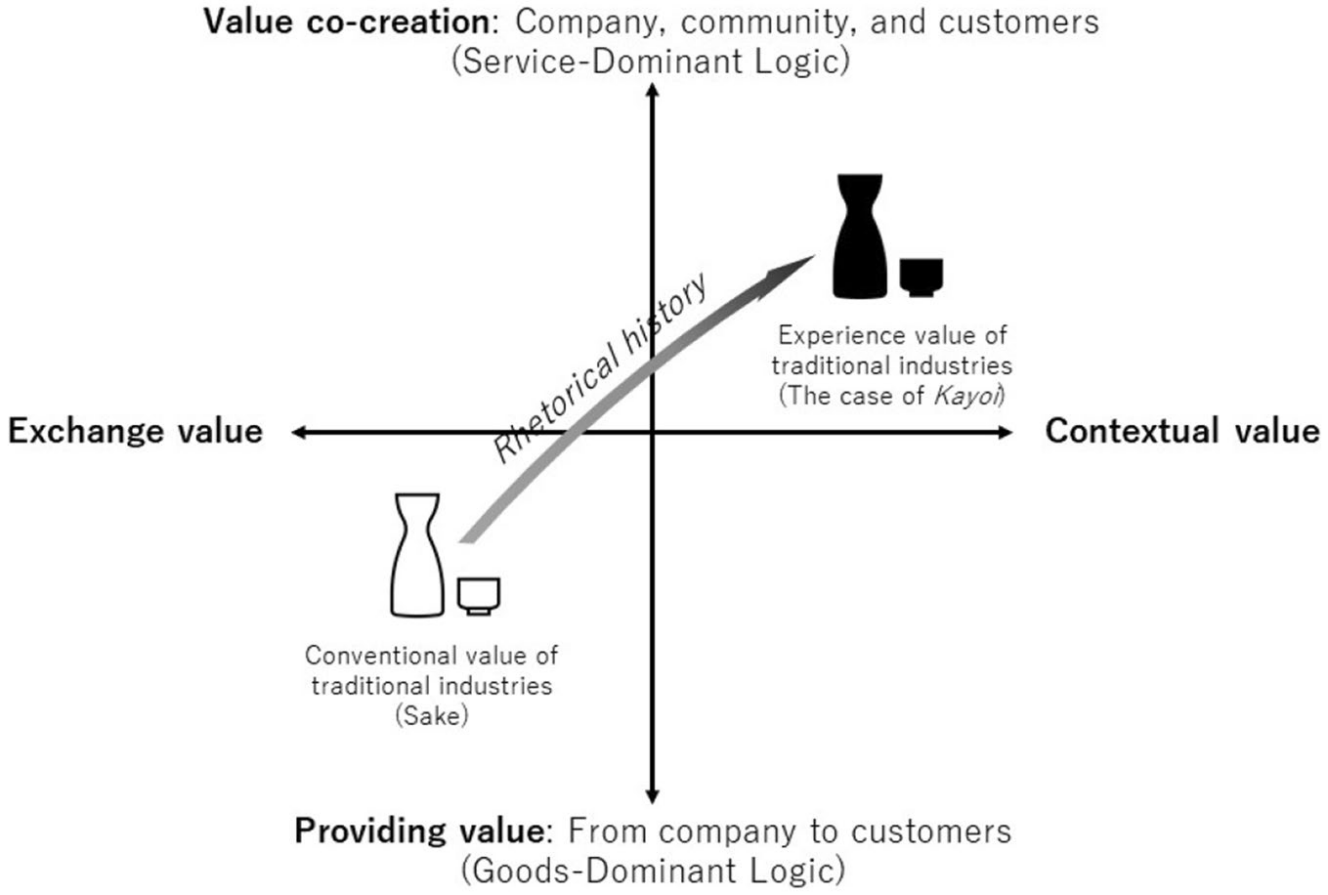
Value expansion of tradition-bound products

This study has managerial implications for constrained industries such as traditional products that cannot grow linearly through premiumization and differentiation as in the experience economy (Pine and Gilmore 1999). It is a detour; however, it shows the advantages of using rhetorical history and simultaneously aiming to form a new ecosystem. In essence, it is a way of looking back, but not simply returning to the past. Instead, it requires reinterpreting history in a modern way, forming new ecosystems, and developing them in a nonlinear manner while co-creating new value with customers and other stakeholders (Fig. 4).

We believe that this conclusion is applicable to the revitalization of other tradition-bound products and industries as well. A company’s history, as well as the history and attachment to a product category, can serve as a mediator. Moreover, empathy for superior design and craftsmanship can serve as a motivation for customers to participate in value co-creation. In this way, a product tied to its own history can co-create value with customers and local resources without compromising its own history while also utilizing new technologies and thus riding a trend that emphasizes experiential consumption (e.g., Morewedge et al. 2021).

Furthermore, this case study provides a significant clue to environmental, social, and governmental management. It shows how customers were attracted to the design of the stainless-steel bottles. They started thinking of the bottle as “My Bottle” and used it repeatedly, thereby reducing the amount of waste. It helped them contribute to local companies and environmental issues. It corresponds to the work on the 12th sustainable development goal, “Responsible Consumption and Production.” Coincidentally, this brewery delivers quality sake to customers, especially for those who wanted contactless service during the COVID-19 pandemic.

## Conclusion

As suggested in the introduction, this theoretical framework helps us understand how to overcome the adverse aspects identified in existing rhetorical histories (e.g., Aeon and Lamertz 2021). Furthermore, it provides useful insights for storytelling (e.g., Mora and Moscarola 2010; Mora and Livat 2013; Lee and Shin 2015) and retro-marketing that seeks to connect brands and consumers more deeply and emotionally (e.g., Gajanova and Zdenka 2020; Pİr 2019).

Many companies are struggling to keep their traditions alive. Some try to develop horizontally by adopting other cultures and trends. This study insisted on the use of rhetorical history to enable value co-creation in a new ecosystem by engaging consumers and local communities without eliminating the essence of tradition. The process of co-creating value by empathizing with the authenticity of history dissolves the boundary between consumers and firms**,** and consequently, consumers become advocates. This is what drives the value embedded in tradition-bound products to vertically increase their social status.

## Research limitations and suggestions for future research

This study has some limitations. Since this study took a case study approach, it may not be generalizable, even though it is probabilistic. Nevertheless, we argue that there is a possibility that we can generalize this theoretical framework to other traditional industries rooted in history and culture, which implies the need for empirical research for further theory building. Therefore, we would like to suggest that future research should focus on building and elaborating a theory of empirical economy, co-creation, and ecosystem of traditional industries based on rhetorical history, which we suggested in this study (Eisenhardt 1989; Sato 2016).

How rhetorical history is effectively used on a case-by-case basis is a crucial issue for future research. Throughout this study, we argued that the strategic use of rhetorical history can potentially shift outcomes—such as the transformation from authenticity to identity. However, the amplification, reduction, and reorganization of meaning and spurious history use have not been examined in strategic operations. The degree of value co-creation and relationship between the two require further study.

## Data Availability

Data sharing and data citation are encouraged.
